# Genetic and Cyto-Histological Analyses in *Olea europaea* L. Cultivars in Parent–Child Kinship

**DOI:** 10.3390/ijms27010094

**Published:** 2025-12-22

**Authors:** Maria Eugenia Cáceres, Luigi Russi, Marilena Ceccarelli, Mauro Mazzocchi, Federico Pupilli, Nicolò Cultrera

**Affiliations:** 1 Institute of Biosciences and Bioresources (IBBR), National Research Council, 06128 Perugia, Italy; mariaeugeniacaceres@cnr.it; 2 Department of Agricultural, Food and Environmental Sciences, University of Perugia, 06121 Perugia, Italy; luigi.russi@unipg.it; 3 Department of Chemistry, Biology and Biotechnology, University of Perugia, 06123 Perugia, Italy; 4 Institute of Science, Technology and Sustainability for Ceramic Materials-National Research Council of Italy (ISSMC-CNR), 48018 Faenza, Italy; mauro.mazzocchi@issmc.cnr.it (M.M.); federico.pupilli@issmc.cnr.it (F.P.)

**Keywords:** *Olea europaea* L., Frantoio, FS-17, Don Carlo, Ascolana Tenera, consanguinity, cyto-histological analyses, morphometric analyses, fruit development

## Abstract

Modern olive breeding points to a plant model characterized by low vigour, high productivity, and resistance to biotic and abiotic stresses, all traits required by the intensive and superhigh-density (SHD) systems of olive tree growing. The Italian Don Carlo and FS-17 Favolosa stand out among the new cultivars that are being tested. They were obtained not by breeding but by mass selection from two seedling populations of the Frantoio cultivar (maternal parent). Here, a multidisciplinary approach was used to determine the paternal parent of Don Carlo and FS-17, and then to investigate the inheritance of interesting traits such as fruit cell dimensions and oil content in these cultivars. Microsatellites were applied in phylogeny and kinship analyses, along with two functional markers previously developed on *OeACP1* and *OeACP2* genes. Ascolana Tenera cultivar was identified as the paternal parent of both new cultivars. This result was also supported by the analysis of the self-incompatibility group of the new cultivars and their most likely paternal parents. Light and electron microscopy [Cryo Scanning Electronic Microscopy (CRYO-SEM), Electronic Scanning Microscopy (E-SEM), and Transmission Electron Microscope (TEM)] techniques were used to analyze the fruit development concerning oil accumulation. Significant differences in cuticle thickness, size and shape of mesocarp and exocarp cells, and oil content were detected among cultivars. Our results suggested that the rearrangement of the traits studied led to an improved progeny compared to the parents. FS-17 exhibited an oil storage efficiency higher than Frantoio. Don Carlo showed fruit traits and oil content almost intermediate between the parents, making it a dual-purpose cultivar.

## 1. Introduction

*Olea europaea* L. subsp. *europaea* is one of the most important crops in the Mediterranean Basin, due to its ecological, economic, and cultural value [1,2]. Its cultivation, which accompanied the emergence of Early Mediterranean civilizations [3,4], inevitably has undergone changes over time. Starting in the 1960s, intensive and later superhigh-density (SHD) systems for olive tree cultivation expanded across the Mediterranean Basin, allowing for good mechanized agronomic management and high productivity in fruits and oil. The demand for increased olive and oil production is rising, and germplasm knowledge and improvement can play a key role in guiding production agriculture towards this sector’s sustainability. Modern objectives for olive breeding point to a new plant model characterized by low vigour, constant and high fruit set capacity, and tolerance to cold, salinity, drought, diseases, and pests, as well as a reduced juvenile period, which is a limiting factor in breeding progress [5]. None of the traditional cultivars, which mainly originated from primary domestication and selection [2,6], possess all of these desirable traits that could be introgressed into cultivars by conventional breeding [7]. For these reasons, many selection programmes were started in the second half of the twentieth century [5] to obtain new varieties that meet the current goals of the olive industry. Several cultivars have been patented, such as the Israeli Askal [8], the Spanish Siquitita (formerly Chiquitita) [9], the Sultana [10], and the Italian Lecciana [11]. Still, the National Research Council (CNR) patented some cultivars in Italy. FS-17 Favolosa [12] and Don Carlo [13,14] stand out. Both were obtained by mass selection from two seedling populations of the cultivar Frantoio. FS-17 Favolosa (hereinafter, FS-17) that originated from open pollination and was initially developed as a clonal rootstock; Don Carlo originated from self-pollination [15]. The Don Carlo cultivar is characterized by high parasite resistance, a good pulp-to-stone ratio, excellent fruit size, and suitable organoleptic qualities, making it a dual-purpose variety. FS-17 is a low vigour cultivar in the adult phase, and is self-pollinating, with an early and abundant productivity. Its fruits, of medium size, have a high pulp-to-stone ratio, showing a high oil yield and an excellent oil quality (cf. patents released). Moreover, this cultivar is highly resistant to *Xylella fastidiosa* subsp. *pauca* (ST53) [16], which causes Olive Quick Decline Syndrome [17,18], negatively affecting oil production in Puglia, one of the most productive regions of Italy regarding olives and olive oil [19]. Both cultivars are being tested in experimental SHD orchards [20].

Given their importance in olive growing, we found it interesting to know the male parent of FS-17 and verify the parental origin of Don Carlo. The knowledge of both parents of an offspring is useful when studying the segregation of specific genetic and phenotypic traits in more depth. This aspect is still lacking for traditional olive cultivars, which were obtained by empirical selection, without knowledge about their origins or the genetic control of interesting agronomic characteristics.

In olives, the inheritance of traits related to oil content or resistance to biotic and abiotic stresses in the progeny of preferential crosses has been studied by several approaches, including genetic, phenotypic [21,22], phenological [23], morphological [24], biochemical [25], and evolutionary [26] analyses.

Variations in allele frequencies over time can provide insights into the effects of genetic drift, natural selection, and gene flow in driving evolutionary changes [27,28]. Molecular markers such as simple sequence repeats (SSRs) or single-nucleotide polymorphisms (SNPs) have been used in parentage analyses and in estimating kinship relationships among olive cultivars, e.g., [29,30], as well as to investigate genetic selection patterns and assess the loss or gain of genetic variability in cultivated populations of different species [31,32,33,34]. Moreover, these markers help identify advantageous mutations affecting the complex distribution of genomic blocks passed on to future generations [35,36].

Fingerprinting and kinship analyses based solely on neutral markers, such as SSRs [21,37,38,39], show several limitations due to genotyping errors and potential mutations in highly variable loci. These issues can reduce the discriminatory power in gene pools that have undergone strong bottlenecks [40]. However, when SSRs are applied along with functional markers [36,41], they become a powerful and reliable tool for molecular characterization, assessment of consanguinity degree, or trait inheritance studies in plant populations, due to their high diagnostic efficiency and independence from environmental variables [42]. By synchronizing genotype data with genetic relationships within an olive germplasm and direct parental lines, researchers can determine whether empirical or targeted genetic improvement, independent of natural population drift, can drive germplasm evolution already at the F_1_ phenotypic level [43].

Olive is a species with a low genetic turnover, and, therefore, breeding programmes should take into account that many cultivars show a self-incompatibility (SI) sporophytic system, also common to some *Olea*-related genera [44,45,46]. This system is controlled by a single locus (S) with only two alleles (S2 allele dominant over S1 allele), and is, therefore, classified as a diallelic self-incompatibility (DSI) system. Only two SI genotypes have been recognized, and olive cultivars can be classified only into two incompatibility groups, G1 (S1S2) or G2 (S1S1), such that cultivars are incompatible within groups and compatible between groups [44]. However, some cultivars can show pseudo-self-compatibility in particular conditions [45,46].

Olive cultivars differ in phenotypic traits of the fruit and oil characteristics. These aspects are closely related to fruit development. Olive drupe is characterized by an exocarp or epicarp covered by a tri-layered cuticle [47,48,49], a fleshy mesocarp (pulp), and a hard endocarp or stone [50]. Fruit development and ripening in olives have long been investigated. These processes are under strict genetic control but are also affected by several environmental conditions [51,52]. The process occurs in four–five months and has been divided into five main stages [53]. Fruit tissues originate from multiple cycles of cell division, enlargement, and differentiation [54,55]. The oil accumulation process in the parenchyma cells of the olive mesocarp, which mainly occurs at the penultimate stage of fruit development, has also been well described. Oil droplet formation starts in localized cytoplasmic regions. These droplets are closely associated and fuse, forming a larger oil droplet that protrudes against and indents the vacuolar membrane. As oil accumulation progresses, new oil bodies appear and fuse with the previously formed ones, resulting in a single oil droplet per mature cell [56,57,58].

In this study, a multidisciplinary approach was applied to identify the paternal parent of FS-17 and verify the origin of Don Carlo by self-pollination of Frantoio. The male parent search was carried out within a wide set, including the olive cultivars in the experimental field, when Don Carlo and FS-17, and others from the Mediterranean Basin, were selected. Genetic relationships among the cultivars were studied by applying neutral (SSRs) and functional [36] markers. In addition, to evaluate the segregation of phenotypic traits in the parent–offspring kinships, cyto-histological and morphometric analyses on the developing fruits were carried out using different light and electron microscopy techniques.

## 2. Results

### 2.1. Genotyping and Phylogenetic Analysis

**Figure 1:**
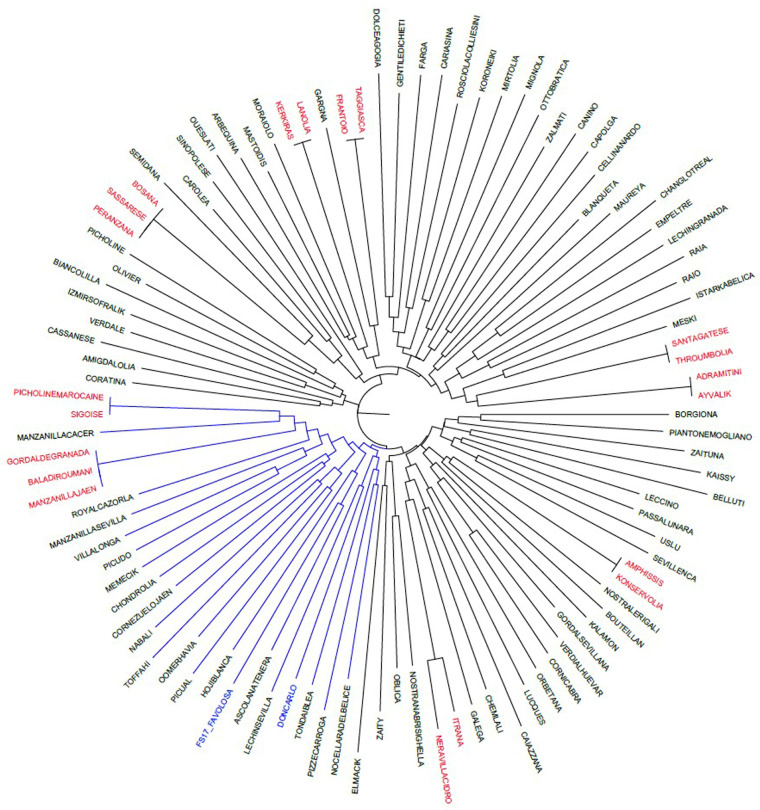
Neighbour Joining phylogenetic tree showing the relationships among the 102 cultivars genotyped with twelve molecular markers. The sub-branch 3A, including Don Carlo and FS-17, is in blue. Synonym cultivar names are in red.

Don Carlo and FS-17 olive cultivars were genotyped, for the first time, with ten ranked SSRs and two functional markers. Their genetic relationships were first investigated by Neighbour Joining (NJ) analysis within a wide set of cultivars, including Umbrian and other Mediterranean cultivars already genotyped (Figure 1).

The phylogenetic tree obtained by using twelve molecular markers harboured three main branches (Figure 1). It allowed for a more specific analysis of the cultivar relationships compared to that obtained by only applying the ten neutral SSRs. Not all cultivars were grouped in structured clusters according to their geographical origin. Indeed, the first branch included most of the Italian cultivars (22), several Greek cultivars (8), admixed with a few Spanish ones (6), two Tunisian cultivars, and one Turkish cultivar. The second branch included a few, primarily French and Italian, cultivars. The third branch, split into sub-branches 3A and 3B, mainly included Spanish and Italian cultivars (3A), and cultivars regionally admixed (3B).

Regarding the two cultivars under study, Don Carlo and FS-17 clustered in sub-branch 3A, a bit distant from their maternal parent, Frantoio, clustering in the first branch. Four other Italian cultivars, namely Tonda Iblea, Nocellara del Belice, Pizz’e Carroga, and Ascolana Tenera, are all characterized by a medium–large fruit and a dual-purpose attitude (*i.e*., oil and table olives), and are clustered in sub-branch 3A. Their molecular profiles were aligned with those of Don Carlo and FS-17. Interestingly, only Ascolana Tenera shared 50% of its genetic profile with both FS-17 and Don Carlo. Table 1 shows the genetic profiles of Don Carlo and FS-17, along with those of Ascolana Tenera and the maternal cultivar Frantoio. By comparing the allele profiles of the new cultivars with those of the putative parents, an almost perfect correspondence in allele length is found, with the only exception being allele 165 for DCA18. Both of the new cultivars are heterozygous at most loci, except for DCA5, EMO90, and *OeACP1* in Don Carlo, and for GAPU71b and GAPU101 in FS-17.

Moreover, NJ analysis confirmed all the synonymies included in the analyzed set (i.e., Adramitini/Ayvalik, Throumbolia/Santagatese, Frantoio/Taggiasca, Lanolia/Kerkiras, and Bosana/Sassarese/Peranzana, in the first branch; Sigoise/Picholine Maroccaine and Baladi Roumani/Gordal de Granada/Manzanilla de Jaen in sub-branch 3A; and Nera di Villacidro/Itrana and Konservolia/Amphissis in sub-branch 3B).

### 2.2. Parentage Analyses

Kinship relationships within the entire set of cultivars were more explicitly investigated by means of COANCESTRY, by using the same set of markers.

The cumulative frequency distribution of inbreeding values (Table S1), across all cultivars and regardless of their geographical origin (Table S2), showed that out of a total of 5151 dyads, 2136 (41.47%) were unrelated. Dyads identified as second and first cousins were 1667 (32.36%) and 800 (15.53%), respectively. A total of 419 dyads (8.13%) were classified as siblings, uncles, or as having grandparent–grandchild relationships (0.1995 ≤ r ≤ 0.379; Table S1), while only 116 dyads (2.25%) were in direct consanguinity (parent–child or full siblings; 0.4003 ≤ r ≤ 0.6384; Table S1). Thirteen dyads (0.25%) were formed by cultivars showing the same identity (0.9472 ≤ r ≤ 1; Table S1), confirming the synonymies highlighted by NJ analysis (Figure 1).

Cultivars involved in the main kinship relationships (half-sibling/uncles/grandparent–grandchild category and parent–child/full sibling category) were 99 and 73, respectively (Table S1). The latter group included FS-17, Don Carlo, Leccino, Ascolana Tenera, and the synonymy Frantoio/Taggiasca (Tables S1 and S3 and Table 2).

FS-17 and Don Carlo were involved in four and three dyads, respectively. The cultivar engaged in the highest number of dyads (18) was Gordal Sevillana, followed by Lechín de Granada, the synonyms Sigoise/Picholine Maroccaine, and Royal de Cazorla, each with eight dyads (Table S3).

The genetic profiles were aligned to identify the closest parent–child relationships within the dyad groups involving the two new cultivars. The direct consanguinity of both FS-17 and Don Carlo with their maternal parent Frantoio was confirmed (TrioML r-values of 0.4605 and 0.4758, respectively; Table S1 and Table 2). The dyads formed by Ascolana Tenera and FS-17 or Don Carlo showed the highest r-values (0.500 and 0.4856, respectively; Table S1 and Table 2). It has to be noted that FS-17 also showed consanguinity with the Leccino cultivar. However, analyses using the specific markers for self-incompatibility groups excluded a paternal role for Leccino. As a matter of fact, both Frantoio and Leccino belong to the same SI-G1 group, while FS-17 and Don Carlo belong to SI-G2, which includes Ascolana Tenera (Table 2). This result, confirming the suggestions from the NJ analysis, led us to consider Ascolana Tenera as their potential paternal parent.

**Figure 2:**
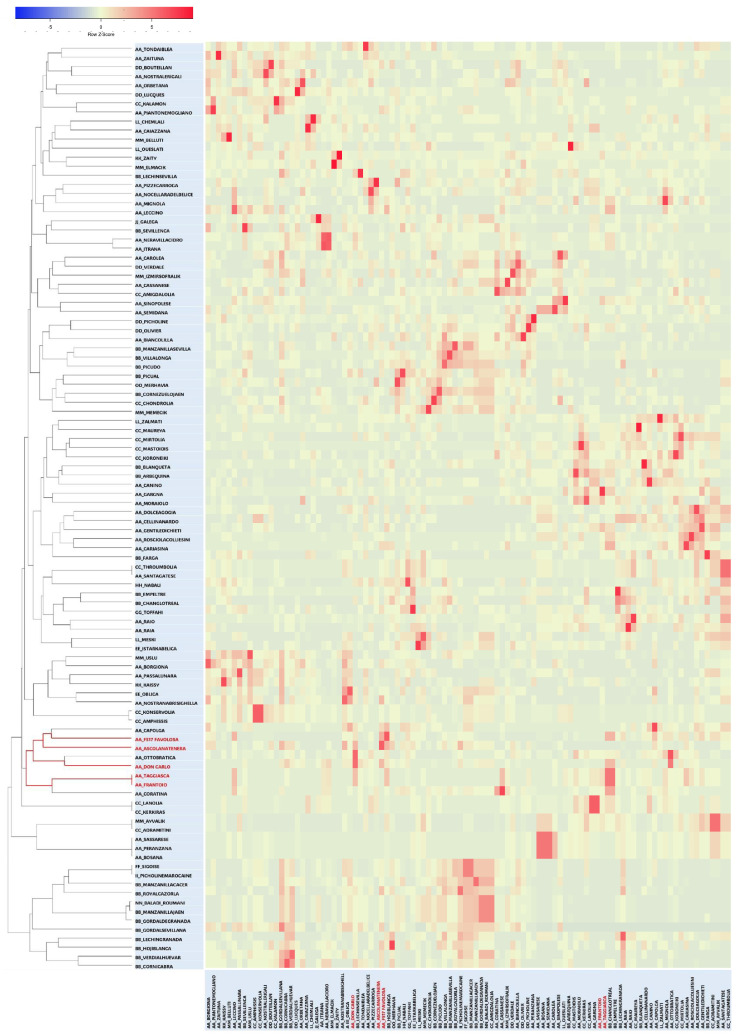
Heat map expression from TrioML correlation r-values triangular matrix based on COANCESTRY dyads, and related kinship tree. Don Carlo, FS-17, Ascolana Tenera, and the synonyms Frantoio/Taggiasca are highlighted in red.

To validate the relationships identified so far, a triangular matrix using the TrioML r-values of the entire genotyping dataset (Table S1) was used to generate an Expression-based COANCESTRY Dyads Heat Map and a related UPGMA tree (Figure 2).

The same matrix was also used in a WARD clustering analysis (Figure S1). The two dendrograms defined a branch confirming the strong kinship of FS-17, Don Carlo, the synonymy Frantoio/Taggiasca, and Ascolana Tenera. The same branch included Capolga and Ottobratica, whereas the Coratina cultivar appeared in the COANCESTRY Dyads UPGMA tree (Figure 2) but not in the WARD tree (Figure S1). Considering TrioML r-values, a direct relationship of Ottobratica, Capolga, or Coratina with the two new cultivars can be excluded (Table S1). Interestingly, the latter showed a second-degree relationship with FS-17 (r = 0.0573) and a first-degree relationship (r = 0.4870) with Frantoio, from which FS-17 is derived (Table S1). The results were corroborated by manually aligning the genetic profiles of the cultivars that clustered together in the phylogenetic trees (Figure 1 and Figure 2), analyzed in all possible three-way combinations to search for direct relationships.

### 2.3. Analysis of Morpho-Cytological Characters

Different light and electron microscopy techniques were used in combination, which have proven to be very effective for studying the development of the fruit tissues in relation to oil accumulation in olive as well as in other species [59].

**Figure 3:**
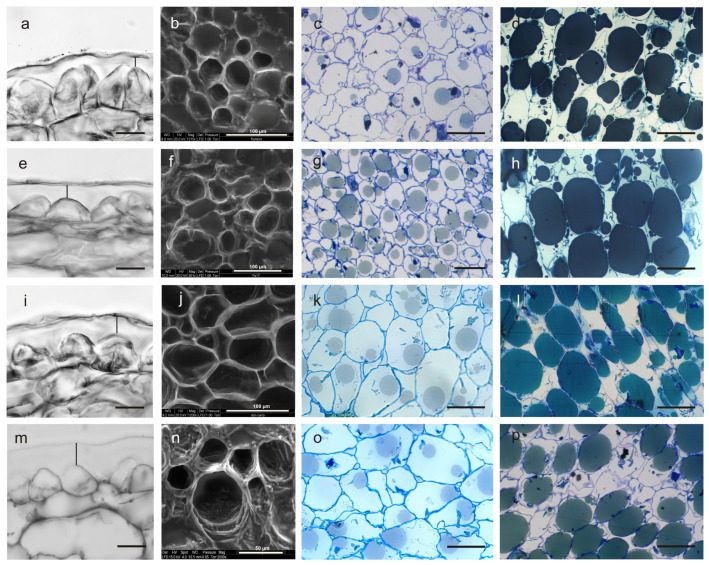
Cross-sections of the olive fruit pulp in cultivars Frantoio (**a**–**d**), FS-17 (**e**–**h**), Don Carlo (**i**–**l**), and Ascolana Tenera (**m**–**p**). Epidermis (exocarp) and cuticle, embedded in paraffin and observed under light microscopy; the vertical black bar shows the cuticle thickness (**a**,**e**,**i**,**m**). Cells of the mesocarp observed under E-SEM (**b**,**f**,**j**,**n**). Cells at stage II (**c**,**g**,**k**,**o**) and stage IV of the fruit development (**d**,**h**,**l**,**p**) in semi-thin sections observed under a light microscope. Scale bars: (**a**,**e**,**i**,**m**) = 20 µm; and (**c**,**d**,**g**,**h**,**k**,**l**,**o**,**p**) = 50 µm. All the sections have been obtained by excision of the drupe in the equatorial zone.

The inner cuticular layer at stage IV of fruit development, predominantly formed by TAG-rich lipoproteins (TRL), is shown in Figure 3a,e,i,m; black bar.

Its thickness was significantly higher in Ascolana Tenera, with a value of about 15.7 µm, higher than that of Don Carlo (12.12) and, in turn, higher than in FS-17 and Frantoio, which showed similar values (7.78 µm and 7.52 µm, respectively) (*p* < 0.01) (Table 3).

The outer pectin layer appeared relatively homogeneous and electron-dense in Frantoio compared to the other three cultivars, particularly to Ascolana Tenera (Figure 3). The epidermal cells of ripening Frantoio fruits were triangular in shape, with their main apex oriented toward the outer layer. In contrast, these cells appeared flatter and dome-shaped in the FS-17 cultivar and even more rounded in Don Carlo and Ascolana Tenera (Figure 3a,e,i,m). Morphometric differences were evident at the two fruit developmental stages (*p* < 0.01), both in fresh pulp sections observed under the E-SEM microscope (Figure 3b,f,j,n) and in paraffin- and resin-embedded sections observed under the light microscope (Figure 3c,g,k,o and Figure 3d,h,l,p, respectively). As expected, the length of epidermal cells increased from stage II to IV (*p* < 0.01). At stage II, the cells in the middle layer of the drupe mesocarp were significantly longer in Ascolana Tenera and Don Carlo (51.75 µm and 51.07 µm, respectively) compared to those of FS-17 and Frantoio, which also slightly differed between them (38.19 µm and 34.24 µm, respectively; *p* < 0.01) (Table 3). Moreover, at the end of stage IV, in the former cultivars, the cell length increased to 62.28 µm and 61.70 µm, respectively; FS-17 and Frantoio showed a comparatively smaller increase in this trait (46.50 µm and 39.84 µm, respectively), and a significant difference between the two values was also observed (Table 3). The mean cell width also increased from stage II (29.49 µm) to IV (35.04) (*p* < 0.01). At stage II, the four genotypes showed significant differences, with a different ranking compared to cell length. Indeed, cells were larger in Don Carlo than in FS-17 drupes, followed by Frantoio and then by Ascolana Tenera (*p* < 0.01) (Table 3). At stage IV, the cell width in the four cultivars maintained the same ranking and differences, but Ascolana Tenera and Frantoio showed similar values (Table 3).

The ANOVA on cell length-to-width ratio showed no differences between stages. Instead, significant differences were found among cultivars whose mean values were similar when passing from stage II to IV, with higher values in Ascolana Tenera, followed by Don Carlo, and, in turn, by FS-17 and Frantoio, showing similar values (*p* < 0.01) (Table 3).

Mesocarp cell surface, measured on both E-SEM and light microscopy micrographs (Figure 3b,f,j,n; Figure 3c,g,k,o; Figure 3d,h,l,p, respectively), highlighted significant differences between the two stages and among all four cultivars (Table 3). The largest cell surface during ripening was detected in Don Carlo drupes (1.278 mm^2^ and 1.744 mm^2^ at stage II and IV, respectively), followed by Ascolana Tenera (0.928 mm^2^ and 1.389 mm^2^, respectively), and then by FS-17 (0.812 mm^2^ and 1.173 mm^2^). The smallest cell area was measured in Frantoio drupes (0.670 mm^2^ and 0.923 mm^2^ at stage II and IV, respectively).

The oil percentage in the fresh mesocarp showed the highest value in FS-17 (18.78%), followed by Frantoio and Don Carlo (both around 15.6%), and then by Ascolana Tenera, exhibiting the lowest value (10.17%; *p* < 0.001) (Table 3).

**Figure 4:**
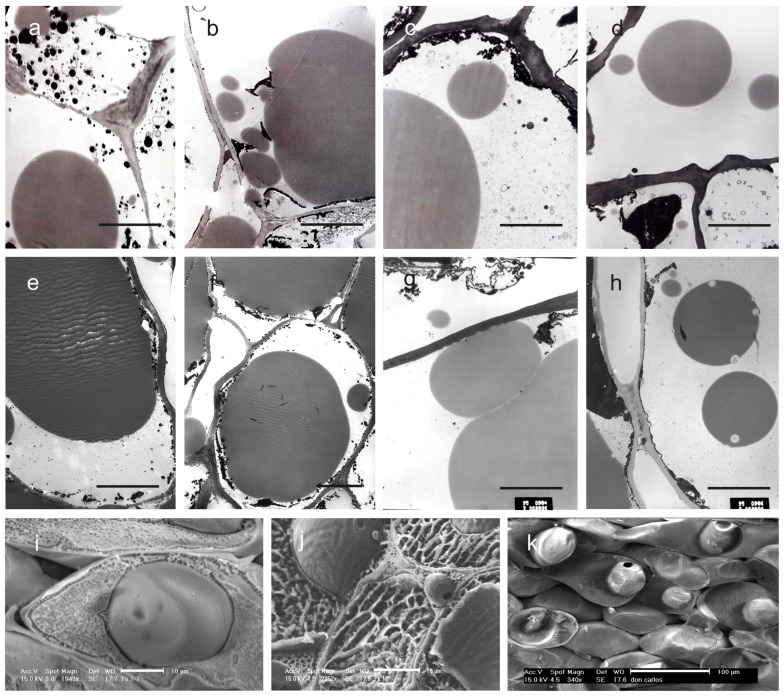
Thin sections of the mesocarp at stages II (21 DAF) and IV (150 DAF) of the olive fruit development observed under TEM (**a**–**h**) and CRYO-SEM (**i**–**k**). All the sections have been obtained by excision of the drupe in the equatorial zone. The micrographs are related to cultivars Frantoio (**a**,**e**), FS-17 (**b**,**f**), Don Carlo (**c**,**g**), and Ascolana Tenera (**d**,**h**). (**a**–**d**) = stage II; (**e**–**h**) = stage IV. CRYO-SEM images have been obtained on drupes at stage IV. Micrographs show oil bodies located in the cytoplasm of the mesocarp cells in FS-17 (**i**) and Don Carlo (**k**), and into the intercellular spaces in the mesocarp of FS-17 drupes (**j**). Scale bars: (**a**–**j**) = 10 µm; and (**k**) = 100 µm.

The formation of the oil droplets in the mesocarp cells was observed by light and CRYO-SEM microscopy. This technique ensures that oil is not lost from the samples because it does not require cycles of organic chemical replacements, which is usually necessary to embed samples in paraffin or resin. Moreover, the subcellular components remain intact [56]. Oil accumulation started at stage II (Figure 4a–d).

The process started early in FS-17, followed by Don Carlo, Ascolana Tenera, and Frantoio (Figure 3c,g,k,o). From our observations, it was possible to state that small and homogeneous oil droplets formed simultaneously in the mesocarp cells and subsequently coalesced to produce a single oil body occupying most of the cytoplasm at stage IV (Figure 3d,h,i,p and Figure 4e–h). Further, CRYO-SEM imaging revealed round oil bodies near and connected with the cell wall (Figure 4j,k). Oil bodies were also observed in the intercellular spaces in FS-17 drupes (Figure 4j), but were never detected in Frantoio or Don Carlo fruits (Figure 4i,k).

## 3. Discussion

By using nuclear microsatellites, the paternal parent of two new Italian olive cultivars, which were very promising for modern olive growing [20], was identified in this study. Don Carlo and FS-17 cultivars were obtained by mass selection from two seedling populations of the cultivar Frantoio. FS-17 was declared to derive from open pollination, whereas Don Carlo was derived from self-pollination [15]. Our results pointed to Ascolana Tenera as the male parent not only of FS-17 but, surprisingly, also of Don Carlo. Moreover, the knowledge of both parents of the two cultivars allowed us to study the distribution of traits such as fruit cell dimensions and oil content, which generally exhibit genotype-specific variations [53,58] in the parents and their progeny. Due to their co-dominant nature, the nuclear SSRs have proven to be a valid tool for kinship analyses. However, it is known that they can also be subjected to mutation via slip-strand mispairing in germ cells during DNA replication [60]. Here, they were initially used to define the genetic profile of Don Carlo and FS-17, and to analyze their genetic relationships with several olive cultivars. The ten ranked SSRs [42] confirmed their discrimination power, also highlighting already known synonymies [36]. In our phylogenetic analyses, the information obtained from these markers was not sufficient to clearly point out the putative parents of the two cultivars, as they clustered in a different branch of the NJ tree from that including the maternal cultivar Frantoio (Figure 1). The additional use of two functional markers, identified within genes controlling the phenotypic traits studied, allowed for a better definition of the phylogenetic relationships of the two new cultivars. Helpful suggestions about the candidate pollinators were gained by focusing on the cultivars clustering with Don Carlo and FS-17. The comparison of their molecular profiles showed Ascolana Tenera as a possible paternal parent of both the new cultivars, excluding that Don Carlo originated by Frantoio self-pollination, as initially supposed.

Parentage analysis by COANCESTRY, which valorised the use of the twelve molecular markers here applied, confirmed Ascolana Tenera as the male parent of the two new cultivars. Don Carlo was involved in dyads of the parent–child type with Frantoio and Ascolana Tenera, whereas FS-17 was involved with Frantoio, Leccino, and Ascolana Tenera. The presence of Frantoio among the relatives of the new cultivars confirmed its role as maternal parent. Regarding FS-17, either Ascolana Tenera or Leccino could be hypothesized as the paternal parent. Molecular checks for self-incompatibility group membership helped exclude Leccino as a possible direct parent. Indeed, it belongs to the same G1 incompatibility group as Frantoio (Table 2) [61], and it is known that cultivars belonging to the same SI group cannot fertilize one another [44]. Aside from that, Leccino and Frantoio resulted in first-degree relatives (siblings; r = 0.4807), thus confirming the previously known relationship. Indeed, Frantoio was classified as a secondary founder for a group of Italian olive cultivars, including Leccino [41].

The alignment of the molecular profiles of Don Carlo and FS-17 with those of Frantoio and Ascolana Tenera showed that the new cultivars inherited one allele of each marker from each parent, except for the DCA18 SSR in Don Carlo (Table 1). This marker has likely undergone a sporadic mutation, producing a 165 bp allele, which is shorter than the common alleles 173, 177, or 179 bp found in this study.

Two findings supported the goodness of kinship relationships obtained in the analyzed set. First, all known cases of synonymy/identity between the cultivars included in this study have been confirmed. Moreover, the cultivar involved in the highest number of dyads in the parent–child/siblings group resulted in Gordal Sevillana, which has been considered the progenitor/founder, via empirical selection, of several traditional Mediterranean cultivars [40,41]. In this regard, it should be noted that the same cultivar is present in the Mediterranean area under different synonyms (Giarraffa, Bella di Spagna, Pizzo di Corvo, and Santa Caterina in Italy, Grosse du Hamma in Algeria, Boube in France, Ters Yaprak, and Tavsan Yuregi in Turkey) [41].

Until a decade ago, little was known about the genetic control of important agronomic traits because, as previously reported, the traditional olive cultivars resulted from empirical selections. The introduction of GBS techniques prompted GWAS studies of traits such as leaf length, whole fruit and stone weight [62,63], fruit growth, oil content, and plant vigour [64]. The availability of whole genome assemblies and annotations of *O. europaea* subsp. *sylvestris* [65], *O. europaea* subsp. *cuspidata* [66], and the genome sequence of several common olive tree cultivars [67,68,69,70,71] significantly contributed to a better understanding of the genetics and genomics in this species and, among others, of fruit morphological traits during ripening and of oil accumulation [72]. In this study, the comparison of cell dimensions and oil accumulation in fruits of the two new cultivars and their parents suggests that the rearrangement of the traits studied led to an improved progeny with respect to the parents.

Morphological analyses on epidermis and mesocarp tissues highlighted peculiar differences in cell shape and size among our cultivars. The new cultivars showed almost intermediate values between those of their parents for cuticle thickness, length of epidermal cells at both the developmental fruit stages studied, and cell length/width ratio (Table 3). The cuticle is an essential component of the olive drupe due to its role as a biological barrier against pathogenic fungi (e.g., *Spilocea oleagina*) or insects (e.g., olive fly, *Bactrocera oleae*) [47,73]. Chemical composition, morphology, and thickness of the cuticle are genotype-dependent characteristics, as shown by comparing olive cultivars grown in the same environmental conditions [74,75] or by analyzing plants of the same cultivar exposed to different irrigation regimes [76]. In our study, the cuticle thickness trait was segregated among the cultivars. Indeed, in Don Carlo, it showed an intermediate value that was closer to the paternal parent, whereas in FS-17, it showed the same value as the maternal parent (Table 3). The measured values are in line with those reported in other olive genotypes [48,77], although higher values (over 40 µm) have also been reported [75,78]. The higher cuticle thickness in Ascolana Tenera and Don Carlo drupes, compared to Frantoio and FS-17, could explain the good pulp consistency of the fruits for being table olives. This characteristic and the medium–large fruit size confirm that Don Carlo can be considered a dual-purpose variety [13].

Regarding the other parameters measured in our samples, a clear trait segregation was seen for mesocarp cell length and width at stage II and stage IV (Table 3). The regular, increasing length of cells in the intermediate layer of the mesocarp during fruit development had already been reported in Frantoio, FS-17, and Don Carlo when these cultivars were grown in two distinct environments [74]. Our results agree with those in [78], who observed an increase in cell size but not in cell division during the same stages of fruit development. More recent studies showed that olive drupe size is genetically regulated [79] and that the differences among cultivars mainly depend on cell number, although cell expansion is the great driving force of fruit growth [80].

CRYO-SEM technology allowed for revealing morpho-physiological differences related to oil accumulation in the cytoplasm. Physiological processes related to the development of oily bodies and the morphometric features of the cells have been well studied in different species, including olive trees, showing that they are species-specific and clearly dependent on genotype [81,82,83,84,85]. Here, it was possible to observe the development of small cytoplasmic oleosomes in all four cultivars. Interestingly, only in FS-17, intact oil bodies were also observed in the intercellular spaces of the mesocarp at stage IV of fruit development. The detection of extracellular oleosomes is a rare event. Before that, oleosomes were detected in this position only in drupes of *Persea americana* Mill [86], whereas in olive trees, they were observed only during fruit over-ripening, which is very late in the process, when cell walls collapse [87].

Oil droplet size and percentage of mesocarp cells containing oil droplets are significant parameters for estimating the oil accumulation process in the mesocarp [78]. Our observations showed that oil accumulation started at different times in the four cultivars, first in FS-17, then in Don Carlo and Ascolana Tenera, and lastly in Frantoio. It is known that early ripening of the fruits and the related early oil accumulation are under genetic control. However, the processes are affected by environmental factors [88] and cultivation techniques [89]. Since our cultivars grow in the same environment, it can be inferred that the differences detected among them are genetically controlled. At the end of stage IV, when the oil accumulation was almost complete, the oil content percentage of Frantoio was similar to that of Don Carlo, but still lower than that of FS-17. These results are in line with previous studies in which FS-17 and its maternal parent Frantoio were compared for oil production [15,89].

When compared to their parental cultivars, FS-17 and Don Carlo appear as superior varieties. The higher oil content of FS-17, combined with its high agronomic performance and known resistance to *X. fastidiosa* [17], make the new cultivar an improved variety with respect to the maternal parent Frantoio. On the other side, some fruit cell traits and oil content were more pronounced in Don Carlo than in the male parent Ascolana Tenera, making Don Carlo a suitable dual-purpose cultivar. The integrated use of genetic, morphometric, and kinship analyses, here applied, seems to be a useful approach to address breeding programmes, which could also be employed to identify putative genes linked to specific phenotypes.

## 4. Materials and Methods

### 4.1. Plant Material

A total of 102 olive cultivars were analyzed in this study. Leaves from adult plants of Don Carlo and FS-17 cultivars (CNR ISAFOM Collection, Tuoro sul Trasimeno) and eight other olive cultivars (Ascolana Tenera, Dolce Agogia, Frantoio, Moraiolo, Nostrale di Brisighella, Nostrale di Rigali, Raia, and Raio), among which most were cultivated in Umbria, Central Italy (CNR-IBBR Collection, Perugia), were collected for DNA extraction (Table S2). Their DNA molecular profiles, obtained as described below, were compared with those of the other 92 cultivars already genotyped with the same markers [21,36,90,91,92]. Thirty-two were of Italian origin (CNR Collections, Boneggio and Lugnano in Teverina), while 60 were of Mediterranean origin, which were kindly provided by the WOGB IFAPA Collection Cordoba, Spain, and Ag. Mamas, Chalkidiki Collection, Greece (provided by CIHEAM Mediterranean Agronomic Institute of Chania, Greece) (Table S2). Overall, the set included more cultivars than those present in the experimental field when Don Carlo and FS-17 were selected (CNR ISAFOM Collection). Indeed, more cultivars were added, some of which were synonyms, to verify the correctness of the methodological approach used in this study. Moreover, the cultivar Leccino was included as a reference for the genetic profile.

For microscopy analyses, drupes from Don Carlo, FS-17, Frantoio, and Ascolana Tenera plants were harvested during the Summer and Autumn of 2022, at two different developmental stages according to Lavee [53]: stage II (seed development and gradual hardening of the kernel at 21 DAF—days after flowering) and the end of stage IV (mesocarp development and intense oil accumulation at 150 DAF). The fruits were collected from the same mature plants previously genotyped. Three plants per cultivar were chosen as replicates, and two random drupes per plant and per developmental stage were used, for a total of 12 fruits per cultivar.

### 4.2. Genetic Analyses

Genomic DNA was extracted from 100 mg of fresh leaves using the GeneElute Plant Genomic DNA Miniprep kit (Sigma-Aldrich, by Merck KGaG, Darmstadt, Germany), following the manufacturer’s instructions. The purity, integrity, and quantity of the DNA extracts were assessed using the Multiskan SkyHigh Microplate spectrophotometer (ThermoFisher Scientific, Waltham, MA, USA). All DNA samples were then diluted to a 25 ng/µL concentration.

Ten highly polymorphic SSR markers (DCA3, DCA5, DCA9, DCA16, DCA18, EMO90, GAPU71b, GAPU101, GAPU103A, and UDO-043) were chosen based on their discrimination effectiveness [42]. In addition, two nuclear markers, an indel on the *OeACP1* gene and an SSR on the *OeACP2* gene, which were considered functional markers, were used. They were chosen because some SNPs on *OeACP1* and *OeACP2* were found to be related to olive fruit weight and palmitoleic acid percentage, respectively (NCBI accession numbers from KF303531 to KF303534) [36].

All the PCR amplifications were performed in a 25 µL reaction volume containing 25 ng of template DNA, 10× PCR buffer, 2.5 mM of each dNTP, 10 pmol of each primer (direct primer labelled with fluorescent dyes FAM, NED, PET, or VIC), and 2U of DreamTaq^TM^ DNA Polymerase (ThermoFisher Scientific). The PCR System 9600 thermal cycler (Applied Biosystems, Foster City, CA, USA) was used. Amplification conditions were as follows: an initial denaturation step at 95 °C for 5 min; 35 cycles: 95 °C for 30 s, an annealing step at a temperature specific to each locus for 30 s (cf. [42]), and 72 °C for 25 s for nuclear SSRs or 40 s for functional markers; and a final elongation step at 72 °C for 30 min. The resulting PCR products were first visualized on 2% agarose (*w*/*v*) gel electrophoresis and then loaded onto an ABI3130 genetic analyzer (Applied Biosystems). The output data were analyzed using GeneMapper 3.7 (Applied Biosystems).

To avoid any bias due to SSR binning and miscalling for stuttering phenomena, the assignment of SSR allelic lengths was validated by comparison with values obtained in studies on other international olive collections [21,93]. A genetic distance matrix was estimated by GenAlEx 6.501 [94,95]. A Neighbour-Joining phylogenetic analyses was carried out by MEGA X software [96], and the graphical representation of the tree was obtained by FigTree software v. 1.4.4 [97].

### 4.3. Parentage Analyses

The kinship relationships among the 102 cultivars were investigated using the COANCESTRY software package v.1.0.1.9, which implements several methods to estimate the pairwise relatedness between individuals as well as the inbreeding coefficients using genetic marker profiles [98]. The relatedness (r) is based on the triadic probability likelihood estimator (TrioML) on pairwise comparisons. The TrioML, combined with specific momentum estimator parameters, was set at 100 reference individuals and 1000 bootstrap [99,100,101,102]. The r-values, ranging from 0 to 1 (a value equal to ‘0’ indicates unrelated cultivars, whereas a value equal to ‘1’ indicates clones), were used to group the 102 cultivars into six kinship categories (r-values and names of categories are reported in Table S1). All the dyads in kinship were visualized in a heat map graphic [103], and the kinship matrix was then used in a hierarchical clustering procedure using Ward’s minimum variance method [104], an algorithm able to minimize the total within-cluster variance. The cluster analysis was validated by 1000 bootstrap replicates using Past software v. 4.09 [105].

### 4.4. DSI Molecular Analyses

To determine which self-incompatibility group, G1 or G2, the cultivars belonged to, markers revealing the presence/absence of the S-locus were amplified on genomic DNA according to [61]. The primers were built on NCBI *Olea europaea* DSI-A marker genomic sequence accessions OP779749.1 and OP779751.1 [106,107].

### 4.5. Microscopy Analyses

#### 4.5.1. Light Microscopy

Drupes collected at the two developmental stages were excised in the equatorial area [108]. Fragments of 3 × 1 mm in size comprising mesocarp and exocarp tissues were fixed in a mixture of paraformaldehyde:glutaraldehyde (1:1, *v*/*v*) in 0.1M cacodylate buffer (pH 7) [109] for 4 h at 4 °C, then washed with the same buffer and post-fixed in a 2% buffered solution of OsO_4_ for 2 h at 4 °C, followed by dehydration in ethanol and embedding in Epon-Araldite (FLUKA). Semi-thin sections (1–2 μm) were obtained with an ultramicrotome (OmU2, Reichert, Heidelberg, Germany) equipped with a glass blade and stained with 0.5% (*w*/*v*) toluidine blue in 2% NaHCO_3_ [110].

Other fragments obtained as above were fixed in FAA (5 mL formaldehyde, 5 mL acetic acid, and 90 mL of 50% ethanol) for at least 24 h at 4 °C, then transferred in 70% ethanol at 4 °C, and finally, dehydrated and clarified in a series of ethanol:xilene mixtures in several volume ratios. Tissues were then embedded in Paraplast, sectioned at 12 µm width with a Reichert-Jung microtome, and stained with a mixture of Safranine-Fast green [111].

Stained samples were observed under a light microscope (DMRB, Leica, Wetzlar, Germany) and images were captured by an ILCE-7 camera (SONY, Tokio, Japan). In brief, measurements (see below) were carried out on 15 cells per sample, for a total of 180 observations per cultivar (3 plants as replicates, 2 drupes per plant, 2 developmental stages, and 15 cells).

#### 4.5.2. Cryo-Scanning Electronic Microscopy (CRYO-SEM) Analysis

Samples obtained as described above were loaded on a sample holder, blocked with a drop of Tissue-Tek O.C.T. [112], and immediately cryo-fixed by plunging them into liquid nitrogen at −180 °C. The samples were then cryo-transferred into the chamber of GATAN ALTO 2100 (2M Strumenti, Rome, Italy), working at −120 °C, fractured with a cold blade, and finally, transferred into PHILIPS SEMXL 20, working at −90 °C to ensure sublimation of the bulk surface water. Surface frosting was avoided by using a low voltage (3–5 KV) electronic beam. Once sublimation was completed, the samples were coated with gold for 120 s at 2mA in the Gatan chamber (−120 °C). Observations were carried out inside the EM chamber (−120 °C), under a 15–20 KV electron beam.

#### 4.5.3. Electronic Scanning Microscopy (E-SEM) Analysis

Samples of fresh epicarp and mesocarp obtained as above were used for the surface morphology analyses. High-resolution micrographs were obtained using a FEI Quanta 200 Environmental Scanning Electron Microscope (ESEM, FEI Corporation, Eindhoven, The Netherlands).

#### 4.5.4. Transmission Electron Microscope (TEM) Analysis

Sample thin sections (0.08 µm) obtained with an ultramicrotome (OmU2, Reichert, Heidelberg, Germany) equipped with a diamond blade were mounted on uncoated copper grids (200 mesh). The contrast was obtained by adding uranyl acetate and an aqueous lead nitrate solution. Observations were carried out with the transmission electron microscope TEM400T (Philips Electron Optics–FEI Company, Hillsboro, OR, USA).

#### 4.5.5. Measurements and Statistical Analysis

The average fruit moisture was obtained on three replicates of 10 fruits per cultivar. The average oil content was estimated in triplicate samples of about 6 g of fresh pulp according to [113]. Parameters related to oil accumulation were calculated only at stage IV.

Observations on exocarp and mesocarp cells were carried out on the central portion of the drupe, since it was seen that the cell characteristics were similar among the apical, central, and basal zones of the mature fruit [108]. From the mesocarp tissue of each sample, cell dimensions were assessed by measuring length, width, length/width ratio, and area. All quantitative analyses on digital images were performed using ImageJ software version 1.53 [114,115]. Data obtained on paraffin sections were compared with those from the semi-thin sections and the fresh sections photographed by E-SEM. The cuticle thickness of the exocarp cells was only determined in drupes at stage IV and was measured as the distance between the outer edge of the epidermal cell wall and the middle inner limit of the lamellar layer of epidermal cells (48).

All measurements were analyzed by a factorial ANOVA, in a model with cultivars and stage as main factors and fruit nested within plants as replicates. Mean separation of treatments was carried out by the Student–Newman–Keuls test.

## Figures and Tables

**Table 1:** Allelic profiles of Don Carlo and FS-17 cultivars and their parents, Frantoio (in red) and Ascolana Tenera (in blue), obtained for the twelve markers used. Allele length (bp) is reported.

Marker	Frantoio	Don Carlo	FS-17	Ascolana Tenera
DCA3	237–243	237–249	231–243	231–249
DCA5	198–206	206–206	206–208	206–208
DCA9	182–206	206–208	206–208	194–208
DCA16	150–156	126–156	150–154	126–154
DCA18	177–179	**165**–177	173–177	173–177
EMO90	188–194	188–188	190–194	188–190
GAPU71b	124–144	124–144	124–124	124–144
GAPU101	183–199	183–201	199–199	199–201
GAPU103a	162–174	136–174	172–174	136–172
UDO43	176–214	176–212	212–214	176–212
*OeACP1*	333–333	333–333	320–333	320–333
*OeACP2*	389–427	389–390	427–465	390–465

The rare allele 165 of DCA18 SSR is in black and in bold.

**Table 2:** Kinship relationships obtained by means of COANCESTRY software v. 1.0.1.9 and related to the dyads formed by cultivars Frantoio and its synonym Taggiasca, Ascolana Tenera, FS-17, Don Carlo, and Leccino. The following are expressed: dyad number (Dyad), cultivar (Individual 1 or 2), self-incompatibility group membership of the individuals (SI-G), and degree of direct kinship between the individuals 1 and 2 of each dyad (TrioML estimator).

Dyad	Individual 1	SI-G	Individual 2	SI-G	POP ID 1 and 2	TrioML
2596	AA_Frantoio	SI-G1	AA_FS-17	SI-G2	AAAA	0.4605
2726	AA_FS-17	SI-G2	AA_Taggiasca	SI-G1	AAAA	0.4605
2681	AA_FS-17	SI-G2	AA_Leccino	SI-G1	AAAA	0.4767
521	AA_Ascolana Tenera	SI-G2	AA_FS-17	SI-G2	AAAA	0.5000
2611	AA_Frantoio	SI-G1	AA_Leccino	SI-G2	AAAA	0.4807
3656	AA_Leccino	SI-G1	AA_Taggiasca	SI-G1	AAAA	0.4807
2305	AA_Don Carlo	SI-G2	AA_Frantoio	SI-G1	AAAA	0.4758
2366	AA_Don Carlo	SI-G2	AA_Taggiasca	SI-G1	AAAA	0.4758
516	AA_Ascolana Tenera	SI-G2	AA_Don Carlo	SI-G2	AAAA	0.4856

The acronym preceding the name of cultivars indicates the country of origin (see Table S2).

**Table 3:** Cell dimensions, cuticle thickness, and oil content in Don Carlo, FS-17, Frantoio, and Ascolana Tenera fruits, measured at two different developmental stages in the mesocarp and exocarp (cuticle thickness).

	Cell Length (µm)	Cell Width (µm)	Length/Width Ratio	Cell Surface (mm^2^)	Cuticle Thickness (µm)	Oil Content (%)
***Stage II***						
Ascolana Tenera	51.75 a	24.53 d	2.21 a	0.928 b	-	-
Don Carlo	51.07 a	35.42 a	1.45 b	1.278 a	-	-
FS-17	38.19 b	30.17 b	1.27 c	0.812 b	-	-
Frantoio	34.24 b	27.83 c	1.23 c	0.670 c	-	-
**Mean**	43.81 B	29.49 B	1.54	0.920 B		
***Stage IV***						
Ascolana Tenera	62.28 a	31.11 c	2.10 a	1.389 b	15.692 a	10.17 c
Don Carlo	61.70 a	40.42 a	1.51 b	1.744 a	12.116 b	15.58 b
FS-17	46.50 b	35.79 b	1.30 c	1.173 c	7.780 c	18.78 a
Frantoio	39.84 c	32.83 c	1.22 c	0.923 d	7.518 c	15.61 b
**Mean**	52.58 A	35.04 A	1.53	1.310 A	10.780	15.03

Mean values within each stage, followed by different lower-case letters, and between stages, followed by upper-case letters, are significantly different at *p* < 0.01.

## Data Availability

Data will be made available on request.

## Supplementary Materials

The following supporting information can be downloaded at https://www.mdpi.com/article/10.3390/ijms27010094/s1.
