# Supplementary material for: Genetic and Cyto-Histological Analyses in Olea europaea L. Cultivars in Parent–Child Kinship
Source: Int J Mol Sci. 2025 Dec 22;27(1):94. doi: 10.3390/ijms27010094 (PMC12785671; doi:10.3390/ijms27010094)
Supplement: Supplementary file 1 [file ijms-27-00094-s001.zip › Figure S1.pdf]

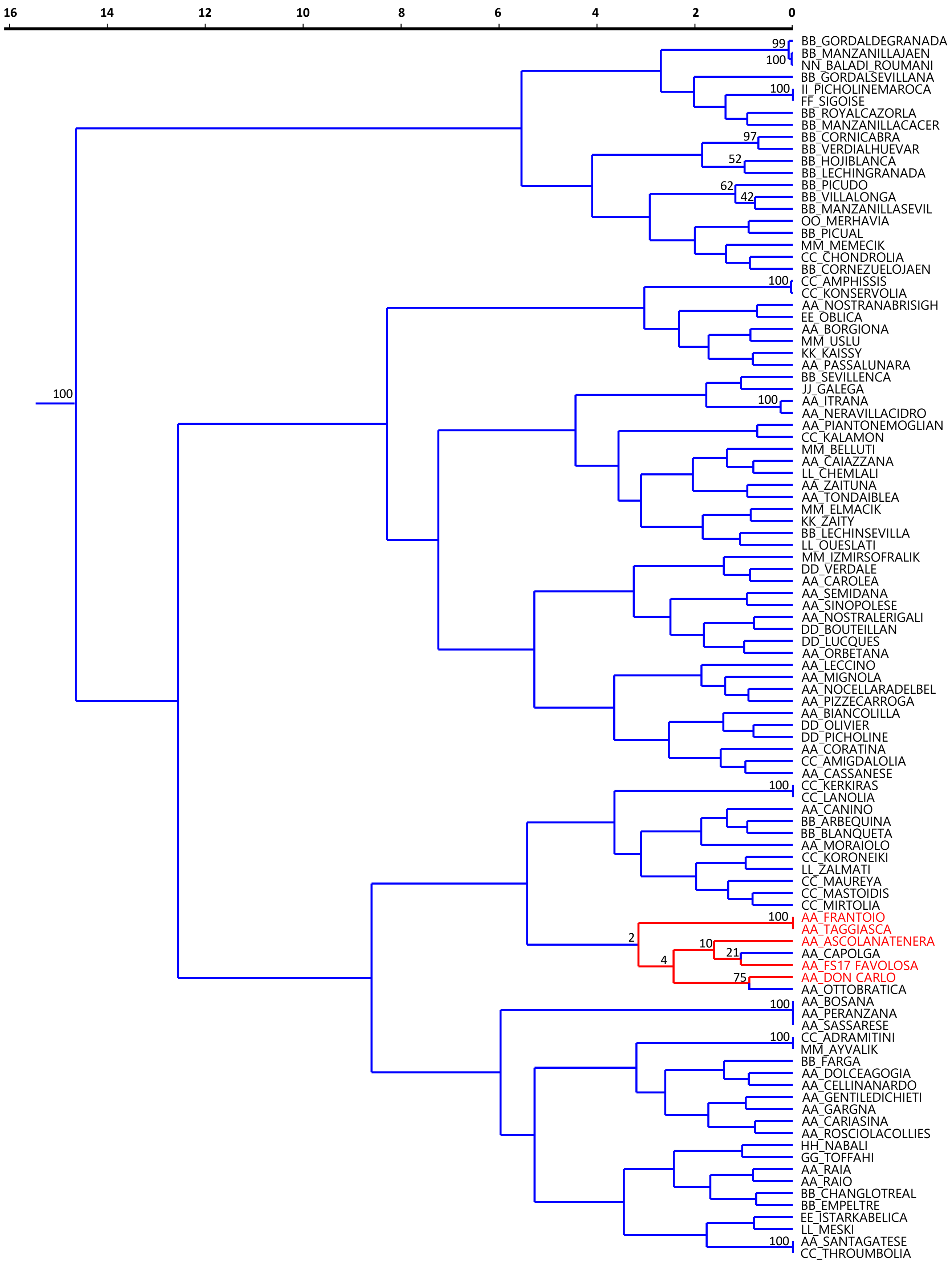

Figure S1. WARDS algorithm clustering tree of the TrioML correlation r-values triangular matrix based on COANCESTRY analysis. Don Carlo and FS-17 cultivars and their parental cultivars are highlighted in red.
