# Supplementary material for: Genetic and Cyto-Histological Analyses in Olea europaea L. Cultivars in Parent–Child Kinship
Source: Int J Mol Sci. 2025 Dec 22;27(1):94. doi: 10.3390/ijms27010094 (PMC12785671; doi:10.3390/ijms27010094)
Supplement: Supplementary file 1 [file ijms-27-00094-s001.zip › Table S2.pdf]

Table S2. List of the olive cultivars investigated, country of cultivation, *ex-situ* collections from which used plants come, and accession. CNR Collection Lugnano in Teverina, Terni, Umbria, Central Italy; CNR-IBBR Collection Perugia, Umbria; CNR core Collection Boneggio, Perugia, Umbria; CNR ISAFOM Collection Tuoro sul Trasimeno, Perugia, Umbria; WOGB IFAPA Collection Cordoba, Spain; Ag. Mamas (Chalkidiki) Collection, Greece. The acronym preceding the name of cultivars indicates the country of origin.

Cultivar	Country of origin	Provenance	Accession
AA_ASCOLANA TENERA	Italy	CNR Collection Lugnano in Teverina	Acc.08C block1
AA_BIANCOLILLA	Italy	CNR Collection Lugnano in Teverina	Acc.12R block1
AA_BORGIONA	Italy	CNR Collection Lugnano in Teverina	Acc.13U block1
AA_BOSANA	Italy	CNR Collection Lugnano in Teverina	Acc.10K block1
AA_CAIAZZANA	Italy	CNR Collection Lugnano in Teverina	Acc.13S block2
AA_CANINO	Italy	CNR Collection Lugnano in Teverina	Acc.07D block1
AA_CAPOLGA	Italy	CNR Collection Lugnano in Teverina	Acc.06C block1
AA_CARIASINA	Italy	CNR Collection Lugnano in Teverina	Acc.10J block1
AA_CAROLEA	Italy	CNR Collection Lugnano in Teverina	Acc.03H block1
AA_CASSANESE	Italy	CNR Collection Lugnano in Teverina	Acc.28N block2
AA_CELLINA NARDO	Italy	CNR Collection Lugnano in Teverina	Acc.09S block1
AA_CORATINA	Italy	CNR Collection Lugnano in Teverina	Acc.10T block1
AA_DOLCE AGOGIA	Italy	CNR Collection Lugnano in Teverina	Acc.13V block1
AA_DON CARLO	Italy	CNR ISAFOM Collection Tuoro sul Trasimeno	Acc.P1 Line 20
AA_FRANTOIO	Italy	CNR Collection Lugnano in Teverina	Acc.13B block1
AA_FS-17 FAVOLOSA	Italy	CNR ISAFOM Collection Tuoro sul Trasimeno	Acc.P1 Line19
AA_GARGNA	Italy	CNR Collection Lugnano in Teverina	Acc.08G block1
AA_GENTILE DI CHIETI	Italy	CNR Collection Lugnano in Teverina	Acc.01D block1
AA_ITRANA	Italy	CNR Collection Lugnano in Teverina	Acc.07E block1
AA_LECCINO	Italy	CNR Collection Lugnano in Teverina	Acc.13F block1
AA_MIGNOLA	Italy	CNR-IBBR Collection Perugia	Acc. M-098
AA_MORAIOLO	Italy	CNR Collection Lugnano in Teverina	Acc.14U block1
AA_NERA VILLACIDRO	Italy	CNR Collection Lugnano in Teverina	Acc.10E block1
AA_NOCELLARA DEL BELICE	Italy	CNR Collection Lugnano in Teverina	Acc.12U block1
AA_NOSTRALE RIGALI	Italy	CNR Collection Lugnano in Teverina	Acc.14T block1
AA_NOSTRANA BRISIGHELLA	Italy	CNR Collection Lugnano in Teverina	Acc.06F block1
AA_ORBETANA	Italy	CNR Collection Lugnano in Teverina	Acc.09F block1
AA_OTTOBRATICA	Italy	CNR Collection Lugnano in Teverina	Acc.31G block2
AA_PASSALUNARA	Italy	CNR Collection Lugnano in Teverina	Acc.12P block1
AA_PERANZANA	Italy	CNR Collection Lugnano in Teverina	Acc.10O block1
AA_PIANTONE MOGLIANO	Italy	CNR Collection Boneggio	Acc.14Line T
AA_PIZZECARROGA	Italy	CNR Collection Lugnano in Teverina	Acc.10D block1
AA_RAIA	Italy	CNR Collection Lugnano in Teverina	Acc.07M block1
AA_RAIO	Italy	CNR Collection Lugnano in Teverina	Acc.14S block1
AA_ROSCIOLA COLLI ESINI	Italy	CNR Collection Lugnano in Teverina	Acc.09D block1
AA_SANTAGATESE	Italy	CNR Collection Lugnano in Teverina	Acc.12K block1
AA_SASSARESE	Italy	WOGB IFAPA Collection Cordoba	Acc. 1306
AA_SEMIDANA	Italy	WOGB IFAPA Collection Cordoba	Acc. 1323
AA_SINOPOLESE	Italy	CNR Collection Lugnano in Teverina	Acc.27M block2
AA_TAGGIASCA	Italy	CNR Collection Lugnano in Teverina	Acc.08I block1
AA_TONDA IBLEA	Italy	CNR Collection Lugnano in Teverina	Acc.12J block1
AA_ZAITUNA	Italy	CNR Collection Lugnano in Teverina	Acc.12E block1
BB_ARBEQUINA	Spain	CNR Collection Lugnano in Teverina	Acc.17Q block1
BB_BLANQUETA	Spain	CNR Collection Lugnano in Teverina	Acc.16F block1
BB_CHANGLOT REAL	Spain	CNR Collection Lugnano in Teverina	Acc.14E block1
BB_CORNEZUELO JAEN	Spain	CNR Collection Lugnano in Teverina	Acc.17C block1
BB_CORNICABRA	Spain	WOGB IFAPA Collection Cordoba	Acc. 10
BB_EMPELTRE	Spain	CNR Collection Lugnano in Teverina	Acc.14D block1
BB_FARGA	Spain	CNR Collection Lugnano in Teverina	Acc.14C block1
BB_GORDAL DE GRANADA	Spain	WOGB IFAPA Collection Cordoba	Acc. 761
BB_GORDAL SEVILLANA	Spain	WOGB IFAPA Collection Cordoba	Acc. 234
BB_HOJIBLANCA	Spain	CNR Collection Lugnano in Teverina	Acc.17U block1
BB_LECHIN GRANADA	Spain	WOGB IFAPA Collection Cordoba	Acc. 54
BB_LECHIN SEVILLA	Spain	WOGB IFAPA Collection Cordoba	Acc. 5
BB_MANZANILLA CACER	Spain	WOGB IFAPA Collection Cordoba	Acc. 430
BB_MANZANILLA JAEN	Spain	CNR Collection Lugnano in Teverina	Acc.18Q block1
BB_MANZANILLA SEVILLA	Spain	CNR Collection Lugnano in Teverina	Acc.18P block1
BB_PICUAL	Spain	CNR Collection Lugnano in Teverina	Acc.18H block1
BB_PICUDO	Spain	CNR Collection Lugnano in Teverina	Acc.18G block1
BB_ROYAL CAZORLA	Spain	WOGB IFAPA Collection Cordoba	Acc. 390
BB_SEVILLENCA	Spain	WOGB IFAPA Collection Cordoba	Acc. 227
BB_VERDIAL HUEVAR	Spain	CNR Collection Lugnano in Teverina	Acc.18E block1
BB_VILLALONGA	Spain	WOGB IFAPA Collection Cordoba	Acc. 364
CC_ADRAMITINI	Greece	Ag. Mamas (Chalkidiki) Collection	Acc.9 MAICH
CC_AMIGDALOLIA	Greece	Ag. Mamas (Chalkidiki) Collection	Acc.12 MAICH
CC_AMPHISSIS	Greece	Ag. Mamas (Chalkidiki) Collection	Acc.7 MAICH
CC_CHONDROLIA	Greece	Ag. Mamas (Chalkidiki) Collection	Acc.6 MAICH
CC_KALAMON	Greece	CNR Collection Lugnano in Teverina	Acc.16V block1
CC_KERKIRAS	Greece	CNR Collection Lugnano in Teverina	Acc.16U block1
CC_KONSERVOLIA	Greece	CNR Collection Lugnano in Teverina	Acc.15U block1
CC_KORONEIKI	Greece	CNR Collection Lugnano in Teverina	Acc.15V block1
CC_LANOLIA	Greece	Ag. Mamas (Chalkidiki) Collection	Acc.1 MAICH
CC_MASTOIDIS	Greece	CNR Collection Lugnano in Teverina	Acc.16S block1
CC_MAUREYA	Greece	CNR core Collection Boneggio	Acc.1 block white
CC_MIRTOLIA	Greece	CNR core Collection Boneggio	Acc.9 block white
CC_THROUMBOLIA	Greece	Ag. Mamas (Chalkidiki) Collection	Acc.16 MAICH
DD_BOUTEILLAN	France	CNR Collection Lugnano in Teverina	Acc.15I block1
DD_LUCQUES	France	CNR Collection Lugnano in Teverina	Acc.15K block1
DD_OLIVIER	France	WOGB IFAPA Collection Cordoba	Acc. 1844
DD_PICHOLINE	France	CNR Collection Lugnano in Teverina	Acc.15L block1
DD_VERDALE	France	CNR Collection Lugnano in Teverina	Acc.15O block1
EE_ISTARKA BELICA	Croatia	CNR Collection Lugnano in Teverina	Acc.17O block1
EE_OBLICA	Croatia	CNR Collection Lugnano in Teverina	Acc.15H block1
FF_SIGOISE	Algeria	CNR Collection Lugnano in Teverina	Acc.17K block1
GG_TOFFAHI	Egypt	WOGB IFAPA Collection Cordoba	Acc. 721
HH_NABALI	Jordan	CNR Collection Lugnano in Teverina	Acc.15P block1
II_PICHOLINE MAROCAINE	Morocco	CNR Collection Lugnano in Teverina	Acc.16I block1
JJ_GALEGA	Portugal	CNR Collection Lugnano in Teverina	Acc.17D block1
KK_KAISSY	Syria	WOGB IFAPA Collection Cordoba	Acc. 789
KK_ZAITY	Syria	WOGB IFAPA Collection Cordoba	Acc. 788
LL_CHEMLALI	Tunisia	CNR Collection Lugnano in Teverina	Acc.18D block1
LL_MESKI	Tunisia	CNR Collection Lugnano in Teverina	Acc.19B block1
LL_OUESLATI	Tunisia	WOGB IFAPA Collection Cordoba	Acc. 114
LL_ZALMATI	Tunisia	WOGB IFAPA Collection Cordoba	Acc. 117
MM_AYVALIK	Turkey	WOGB IFAPA Collection Cordoba	Acc. 97
MM_BELLUTI	Turkey	WOGB IFAPA Collection Cordoba	Acc. 690
MM_ELMACIK	Turkey	WOGB IFAPA Collection Cordoba	Acc. 686
MM_IZMIR SOFRALIK	Turkey	CNR Collection Lugnano in Teverina	Acc.19G block1

MM_MEMECIK	Turkey	WOGB IFAPA Collection Cordoba	Acc. 93
MM_USLU	Turkey	CNR core Collection Boneggio	Acc.19 block white
NN_BALADI_ROUMANI	West Bank	WOGB IFAPA Collection Cordoba	Acc. 1759
OO_MERHAVIA	Israel	WOGB IFAPA Collection Cordoba	Acc. 120
